# Thyroid Hormone Resistance: A 17-Year Follow-up Case Report

**DOI:** 10.2174/0118715303329834240815193640

**Published:** 2024-08-26

**Authors:** Cristina Giusto, Marina Passeri, Patrizia Sperti, Isabella Nardone, Sium Wolde Sellasie, Simona Zaccaria, Lucia Longo, Pietro Lo Deserto, Stefano Amendola, Luigi Uccioli

**Affiliations:** 1 Division of Endocrinology and Diabetes, CTO Andrea Alesini Hospital, Department of Biomedicine and Prevention, University Tor Vergata, 00133, Rome, Italy;; 2 PhD School of Applied Medical-Surgical Sciences, University of Rome Tor Vergata, 00133, Rome, Italy

**Keywords:** Resistance to thyroid hormone, refetoff syndrome, complications, palpitations, hyperidrosis, TSH

## Abstract

**Background:**

Resistance to thyroid hormone is a rare syndrome characterized by peripheral resistance to thyroid hormones. It is caused by genetic dysfunction of thyroid receptor genes, with Thyroid hormone Receptor-beta (TRβ) being the most prevalent. Affected patients show high thyroid hormone levels and non-suppressed Thyroid-stimulating Hormone (TSH). Syndrome manifestations vary from hyperthyroidism to hypothyroidism depending on the specific mutation.

**Case Presentation:**

We, herein, describe the case of a 24-year-old female with a diagnosis of resistance to thyroid hormone from the age of 7. The main symptoms the patients complained about were headaches, palpitations, hyperidrosis, and frequent evacuations with severe underweight. The patient’s blood test showed high FT3 and FT4 levels with a non-suppressed TSH. We performed a disease complications screening that revealed mild osteoporosis and normal cardiac activity (the patient was already treated with bisoprolol).

**Conclusion:**

This case illustrates symptoms and complications of resistance to thyroid hormone syndrome, a rare and misdiagnosed condition. In this case report, we describe and explain long-term disease symptoms and their management. The long-term history of our patient’s disease adds a more comprehensive evaluation of the syndrome and its consequences, contributing to new insights into the resistance to thyroid hormone syndrome and shedding light on personalized management of its manifestations.

## 1 INTRODUCTION

Thyroid hormone regulates an impressive number of genes with a complex and highly regulated pathway that involves tissue-specific thyroid hormone transporters and various Thyroid hormone Receptor (TR) isoforms. The mechanism of Thyroid Hormone (TH) action has been previously studied and is well-known. This report shows the general features of a rare condition known as Refetoff syndrome or Resistance to Thyroid Hormone (RTH) syndrome, characterized by peripheral resistance to TH, due to genetic dysfunction of Thyroid Receptor (TR) genes. This syndrome, with a prevalence of 1 in 40,000 live births, was first described in 1967. Frequency among sexes is identical, while prevalence may vary among different ethnic groups. The transmission is generally autosomal dominant, and rarely caused by mosaicism. There are two TR genes, TRα and TRβ, with differentpatterns of expression in human tissues. TRα is mostly expressed in the brain, heart, and skeletal muscle with three different isoforms. TRβ has three major splice isoforms: TRβ1, expressed in different tissues; TRβ2 expressed in the brain, retina, and inner ear, and TRβ3 expressed in the kidney, liver, and lung [1]. TRα and TRβ present an equivalent DNA structure, but differ mainly in the amino terminus. These variations in the ligand-binding pocket allow specific ligands to interact with TRα or TRβ selectively. TRβ arbitrates thyroid hormone feedback to TRH/TSH, and an alteration of this pathway contrasts this feedback, while in patients with TRα mutations, thyroid hormone feedback is preserved and thyroid hormone levels are not raised, resulting in peripheral hypothyroidism. Patients with TRβ mutations in homozygosis show a more severe phenotype, including goiter, hearing loss, intellectual and growth retardation, and much higher blood ranges of T4, T3, and TSH than heterozygous patients [2]. The most frequent mutations are frame-shift mutations, leading to a non-functional TRβ carboxyl-terminus, which can no longer interact with ligands and cofactors [3, 4]. This syndrome is often misdiagnosed, and clinical manifestations can vary from hyperthyroidism to hypothyroidism [5-7]. In the first situation, the treatment is based on anti-thyroid drugs, while in the case of hypothyroidism, patients should receive L-thyroxine treatment [8]. In conclusion, affected patients show regularly elevated TH levels and non-suppressed ranges of TSH in the absence of acute illness or variations in TH-binding proteins [9]. Other case reports have described the pathogenesis, differential diagnosis, and manifestations of this disease [10]. Our case adds information about the long-term complications of all systems involved and their personalized treatment.

## 2 CASE PRESENTATION

A 24-year-old Caucasian woman with a diagnosis of resistance to thyroid hormone syndrome was admitted to the endocrinological department at CTO Hospital in Rome. When the patient was born at 38 weeks, she suffered from neonatal jaundice and attended phototherapy. TSH was normal and no other investigations were performed. At the age of 7, the patient started experiencing tachycardia, tremors, and delay in psychophysical development. Her blood tests showed high TSH, FT3, and FT4 levels. In order to rule out an organic alteration of the pituitary gland, a pituitary MRI was performed, which resulted negative. A molecular investigation was performed, which resulted in a mutation of the gene encoding for the Thyroid hormone Receptor-beta (TRβ). The patient’s mutation concerned exon 9 and caused a replacement of arginine with tryptophan at codon 338. This mutation was not found in the patient’s mother, father, or sister. Since the diagnosis, the patient was treated with the tri-iodothyronine analogue TRIAC with mild benefits for symptoms, like tremors, and a slight improvement in performance at school. Bisoprolol was added during adolescence since tachycardia was not under control. She reached menarche at the age of 12 and her cycles have always been regular. She experienced some fractures during her childhood; at the age of 10, she broke her right tibia, leading to mild trauma; at the age of 14, she broke her right humerus, accounting for another trauma. From the age of 16, the patient suffered from psychiatric conditions. She was hospitalized multiple times for psychiatric and eating disorders. She was kept on a tight psychiatric follow-up and pharmacologic treatments for anorexia nervosa and major depression, and she attempted suicide at the age of 20. In February 2024, the patient was admitted to the endocrinological department at CTO Hospital in Rome. The main symptoms she complained about were headaches, palpitations, hyperidrosis, and frequent evacuations. After taking the medical history, we decided to hospitalize her in our endocrinological hospital, where disease complications were investigated. Thyroid ultrasound showed normal volume (8.9 ml) with a non-homogeneous echo pattern. The objective examination showed a height of 146,5 cm, a weight of 31 kg, and a BMI of 14.5, revealing highly underweight, with a 64 cm waist circumference. The heart rate was 80 and there were no pathological sounds at the cardiac or lungs examination. There was no abdominal tenderness to palpation, normal rumbling in the bowels, and Blumberg and Murphy signs were negative. Neck palpation showed a normal palpable thyroid without nodules and no lymph nodes were detected. Blood tests revealed TSH 4,84 uUI/ml, FT3 6,38 pg/ml (reference range 1,58-3,91), FT4 1,89 ng/dl (reference range 0,7-1,48), and TPO antibodies 60,7 UI/ml (reference range 0-5.6). The DXA showed a spine Z-score of -2.4, femur neck -3, and femur total -2.1, with a TBS L1-L4 1.262 and normal morphometry. The REMS (Radiofrequency Echographic Multi-spectrometry) was also performed, which showed a spine Z-score of -0.8 and a femur neck -1. The cardiological exam with an electrocardiogram showed normal cardiac activity with a heart rate of 80 and good haemodynamic compensation. The patient was already treated with bisoprolol 1,25 mg, vitamin D, magnesium, lamotrigine 50 mg three times a day, and quetiapine 50 mg once daily. She had suspended TRIAC two years earlier. At the hospital discharge, since the symptoms were well controlled, no therapy was added.

## 3 DISCUSSION

RTHb manifestations can be variable in tissue expression and severity. This variability may be due to the differences in tissue sensitivity to TH and the vast variety of possible mutations [11-16]. The uneven distribution of TRβ and TRα among different tissues is the most likely explanation for this paradox; tissues that express predominantly or only the TRα receptors would respond to the excessive endogenous levels of thyroid hormone, while tissues that express mainly the TRβ receptors would be completely unresponsive to thyroid hormone [8]. Our patient’s mutation, TRβ mutation, is the most frequently found in RTH [5, 7], but the precise incidence of RTHb is unknown. Surveys indicate a prevalence between 1 in 19,000 and 1 in 40,000 live births [3, 12, 13]. At the time of admission to our endocrinological hospital, our patient had 17 years of disease with multiple symptoms, both excess and deficiency of TH. In fact, at birth, the patient suffered from neonatal jaundice, which could have been a sign of hypothyroidism [8]. At 7 years of age, when she was diagnosed with Refetoff S., the patient experienced tachycardia due to the presence of TRα receptors in the myocardium, which retained or increased its sensitivity to TH, and delay in psychophysical development due to TRβ expression in the hypothalamus and pituitary gland, leading to relative hormone deficiency [16, 17]. Afterward, by the age of 16, the patient suffered from psychiatric disorders, with symptoms that could be explained by the predominant expression of TRα receptors in the brain, providing a thyrotoxic local environment. In fact, symptoms, such as “foggy brain” and anxiety, are occasionally reported by RTHb patients, and the treatment strategy can be intermittent high doses of L-T3. In this way, brain function improves due to the reduction in serum T4, a hormone more readily available to the brain and being more thyrotoxic. Another treatment strategy is the use of TRIAC, which has a higher affinity than T3 for several TRβ mutants, but our patient, who was treated with it since her diagnosis, and until 2 years ago, did not report sufficient benefits for her symptoms [3]. Also, significant biochemical improvement was never found in the patient's blood test during her therapy with TRIAC. She also presented at the physical examination with short height despite normal menarche with normal menstrual cycles. Short stature and growth retardation are common features in RTH. In an analysis of 72 individuals, 11 (15%) subjects had a height below the fifth percentile and 6 (8%) below the third percentile, although most measurements were obtained in children, so it was not clear whether they ultimately achieved a normal height as adults [16]. In literature, an improved growth rate in response to thyroid hormone treatment has also been reported [16]. However, our patient in treatment with TRIAC did not reach her target height. She also had osteoporosis with a partial degradation of trabecular bone. As a matter of fact, the major TR isoform in the bone, such as the brain and the heart, is TRα so it responds to the excessive endogenous levels of TH [1]. However, since recent studies show that the DXA could underestimate the Bone Mineral Density (BMD) in short people [15], we also performed REMS. This exam showed non-osteoporotic results, with a Z score >-2. Lastly, in similar cases, one of the most common features is goiter [5] due to TSH stimulation, as the pituitary mainly expresses TRβ. However, our patient’s thyroid was of normal dimension, even with a non-homogeneous echo pattern and TPO positivity. The antibody positivity is in fact possible in RTH due to the chronic thyroid stimulation from high TSH [13, 14] and may require a tighter biochemical follow-up. In conclusion, differences in the disease manifestations are common and not all symptoms can be detected. Different clinical manifestations have been reported in other cases [18]. This case sheds light on the impact of RTH years after its manifestation and suggests considering any patient’s complaint or sign and using it as a call to screen for any disease complication. In conclusion, we recommend frequent psychological and cardiological evaluations with ECG and bone mass screening using new items as well, like REMS, for a safer frequent evaluation.

## CONCLUSION

Today, the patient reports an improvement in her symptoms and is still treated with antidepressants and bisoprolol. The adrenergic and psychiatric symptoms are under control. TRIAC was not introduced again since the thyroid functioning was within limits with a mild elevation in FT3 and FT4 levels. We are considering tighter bone monitoring using the REMS, which in this case could provide a more accurate follow-up and can be used frequently without radiological risks.

## AUTHORS’ CONTRIBUTIONS

All the authors have accepted responsibility for the manuscript's content and consented to its submission. All of them have meticulously reviewed the results and unanimously approved the final version of the manuscript.

## Data Availability

The data that support the case report described will be made available from the corresponding author upon reasonable request.

## LIST OF ABBREVIATIONS

TH: Thyroid Hormone
TR: Thyroid hormone Receptor
RTH: Resistance Thyroid Hormone
TRβ: Receptor-Beta

## STANDARDS OF REPORTING

CARE guidelines were followed.
